# Behavioral responses for facemask use messages to prevent COVID-19 among residents of Bahir Dar City, Ethiopia: an application of extended parallel process model

**DOI:** 10.1186/s12889-022-14872-5

**Published:** 2022-12-22

**Authors:** Tenagnework Eseyneh, Habtamu Wondiye, Zinabu Fentaw, Netsanet Eseyneh, Eyob Ketema Bogale, Hordofa Gutema

**Affiliations:** 1 Department of Public Health, College of Medicine and Health Sciences, Injibara University, Injibara, Ethiopia; 2 Department of Health Promotion and Behavioral Sciences, School of Public Health, College of Medicine and Health Sciences, Bahir Dar University, Bahir Dar, Ethiopia; 3 Department of Epidemiology and Biostatistics, School of Public Health, College of Medicine and Health Sciences, Wollo University, Dessie, Ethiopia; 4 Felege Hiwot Comprehensive Specialized Hospital, Bahir Dar, Amhara, Ethiopia; 5 Department of Health, Behavior and Society, Faculty of Public Health, Institute of Health, Jimma University, Jimma, Ethiopia

**Keywords:** Bahir Dar, Behavioral response, COVID-19, Ethiopia, Extended Parallel Process Model, Facemask, Messages

## Abstract

**Background:**

The coronavirus disease 2019 (COVID-19) pandemic causes major morbidity and mortality in the world. Timely behavioral response assessment of the community is important to shape the next effective interventions and risk communication strategies to adopt preventive behavior. Hence, this study aimed to assess behavioral responses for facemask-use messages to prevent COVID-19 and its predictors among residents of Bahir Dar City, Ethiopia, 2021 by using the Extended Parallel Process Model.

**Methods:**

A community-based cross-sectional study was conducted with the guide of the Extended Parallel Process Model in Bahir Dar city from March 9 to April 9, 2021. A multistage sampling technique was used, and data was collected through a face-to-face interviewer-administered questionnaire using Epicollect5. Descriptive statistics and Binary logistic regression were computed using SPSS V.25. Variable with *P* < 0.25 in the bivariable analysis was a candidate for multivariable analysis to control confounding effect. In multivariable analysis, variables with *P* < 0.05 were considered statistically significant and the result was presented using an adjusted odd ratio (AOR) with a 95% confidence interval (CI).

**Results:**

A total of 616 participants with a response rate of 97.1% were included. Of the total participants, 229(37.2%) were in the danger control response. The behavioral response was affected by Occupational status [AOR (95%CI) 3.53(1.67–7.46)], the number of people living together [AOR (95%CI) 2.62(1.28–5.39)], self-control [AOR (95%CI) 1.14(1.05–1.25)], a friend for the preferred source of information [AOR (95%CI) 5.18(3.22–8.33)] and printed materials for the preferred channel [AOR (95%CI) 2.14(1.35–3.43)].

**Conclusion:**

Above one-third of the participants were in the danger control response. Occupational status, number of people living together, self-control, a friend for the preferred source of information, and printed materials for the preferred channel were independent predictors of resident behavioral response to the use of facemasks. Policymakers should consider students and people who live alone. Message developers should use a friendly person to transmit messages and should prepare printed materials. Activities and strategies should also focus on self-control and perceived efficacy without ignoring the perceived threat.

**Supplementary Information:**

The online version contains supplementary material available at 10.1186/s12889-022-14872-5.

## Background

The new coronavirus disease 2019 (COVID-19) is caused by a severe acute respiratory syndrome coronavirus 2 (SARS-CoV–2) [1]. The World Health Organization (WHO) declared the pandemic of SARS-COVID-19 on March 11, 2020 [2].

The COVID-19 pandemic is one of the topmost modern societal problems, with psychological and socio-economic impacts, and the cause of major morbidity and mortality in the world. On June 18, 2021, there were 178,232,114 cases and 3,858,656 deaths reported globally as of the worldometer COVID-19 weekly epidemiological update [3]. On June 18, 2021, a total of 5,179,703 cases and 136,668 deaths across all of Africa were reported, whereas in Ethiopia, there were 274,775 cases and 4,262 deaths [3]. On the other hand, the Amhara region reported a total of 11,748 cases on June 17, 2021 [4].

The COVID-19 infection may be asymptomatic or acute respiratory disease and the latter may have severe pneumonia, sepsis, and septic shock. There is no specific pharmaceutical management recommended [5]. Both symptomatic and asymptomatic people can transmit the virus to others through respiratory droplets or direct contact [6].

Face mask use, physical separation, frequent hand washing with soap and water, hand rubbing with an alcohol-based sanitizer, and respiratory hygiene are all crucial preventive behaviors that should be followed [7]. Facemasks have been considered a first step to prevent the spread of the disease and could result in a large reduction in the risk of COVID-19 infection. It can also prevent pre-symptomatic transmission during the incubation period [8–10].

COVID-19 requires widespread collective action, positive behavioral responses, and cooperation [11, 12]. The risk communication and community engagement (RCCE) strategic approach was adopted and used in Ethiopia to fight COVID-19 since the first case was reported in Ethiopia [13]. In Ethiopia, there is a strong need to reinforce community awareness and practices to stop the nationwide spread of the virus, but Poor risk communication, fake news, and misinformation could resist the public to adopt protective behaviors and lead to confusion in the public [14].

Even if the government made decisions like lockdown and a state of emergency, it was not strict and has not been controlled the disease [13, 15]. In the presence of many messages distributed to the community through different channels, most people are not practicing the recommended behavior (facemask). Behavioral change to prevent infection is important to control the current pandemic.

A person with poor behavioral responses toward the COVID-19 pandemic was significantly associated with symptoms of psychological distress, depression, anxiety, and insomnia [16]. Studies related to COVID-19 were focused on epidemiology, clinical characteristics, knowledge, attitude, practice, and risk perception [17, 18]. A study done on an online survey in Ethiopia identified that residence, region, religion, and sources of information as predictors for the attitudinal response of COVID-19 prevention messages [19].

Theories and models support describing the process that individuals go through changes as they exchange information, process, interpret and respond to different messages [20]. Theories and models are important to help the selection, development, implementation, and evaluation of interventions along with the planning of health promotion programs [21]. In this study, Extended Parallel Process Model (EPPM) was used. According to EPPM, Behavioral Response is a cognitive or emotional process following a message's recommendations. It is the result of both the perceived threat and perceived efficacy [20].

EPPM has perceived susceptibility, perceived severity, self-efficacy, and response efficacy constructs [20]. EPPM proposes health risk messages induce two cognitive appraisals an appraisal of the threat and an appraisal of the efficacy of the recommended response. Based on these appraisals, one of three outcomes will occur no response, a danger control response, or a fear control response [20]. EPPM tried to clarify when and why recommended message works or fails and to get the category of individuals whether they are in danger control response or not [20, 22].

People must believe COVID-19 is dangerous and that they are vulnerable to it [20]. Furthermore, they must believe that the recommended practice (wearing a facemask) is effective in controlling COVID-19 and that they can perform it to avoid COVID-19. If they perceive both the threat and the efficacy to be high, they readily accept the messages and, as a result, perform the necessary activity to avoid the threat, which is known as danger control (high attitude, intention, belief, behavior change).

If the perceived threat outweighs efficacy, they avoid fear by reducing messages rather than preventing the threat, the response is known as fear control (defensive avoidance, denial, or reactance). Furthermore, if they have a low perceived threat starting from the first appraisal, the people do not operate the message which is called no response [20]. (Supplementary figure S1).

People’s behavioral responses to infectious diseases could control the transmission patterns of disease and the number of new cases [15]. It will be determined by doing research or rapid assessment. This research will solve the above problems and fill the gap in scientific knowledge. Therefore, this study aimed to assess the behavioral responses for facemask-use messages to prevent COVID-19 and its predictors among residents in Bahir Dar City, Ethiopia, in 2021 with the guide of EPPM.

## Methods and materials

### Study design and settings

A community-based cross-sectional study design was conducted from March 9 to April 9, 2021, among residents of Bahir Dar City. Bahir Dar is the capital of Amhara Regional State, which is in the northwestern part of Ethiopia. It is one of the tourist attractions areas in the country, and many people from all over the world came and had contact with the people [23]. During data collection, there was no lockdown. The community, schools, and organizations were on their usual day-to-day activities. According to the Bahir Dar City Municipality office 2019\2020 report, Bahir Dar city has 6 sub-cities, 26 kebeles with a total population of 312,410 from which 145,579 are males and 166,831 are females.

### Population

All residents in Bahir Dar city administration were the source of population. All residents in the selected 8 kebeles during data collection were the study population. Individual ≥ 18 years who resided in Bahir Dar City for ≥ 6 months during data collection were included in the study whereas a person who was critically ill and unable to communicate during the data collection period were excluded from the study.

### Sample size and sampling procedures

The sample size was 634 which was calculated by STATCALC program of Epi-info version 7.2.4.0 statistical package software, based on the single population proportion assumptions that were: A 95% confidence level (Z), 5% margin of error (E), and 50% of the proportion (P) of the Danger control process (because there was no research done on a related topic in Ethiopia previously to the understanding of the principal investigator) and 1.5 Design effect (D).

A multi-stage sampling technique was used to select study households. In the first stage, eight Kebeles were selected from 26 Kebeles using a lottery method by considering the rule of thumb of 30% coverage of representative of the study population. In the second stage, the study households were selected using a systematic random sampling technique considering 21 as the sampling interval. The total number of households was taken from each kebele administration to calculate the sampling interval.

The first household was selected by lottery method from the first 21 households. Then every 21 households started from the first selected household was taken. When each selected household had more than one respondent (study unit), one person was selected by the lottery method at the time of data collection. In the case of non-response after the repeated visit, (two times), the individual was considered as non-response.

$$Kth\;sampling\;interval\;was\;calculated\;as=13,931/634=21$$

### Data collection

A valid data collection tool was adapted from related studies [24–27]. The perception part was based on the risk behavior diagnosis scale (RBDs) approach, adapted to the context of COVID-19 [20, 25, 28]. The RBD is a Likert scale tool that allows rapid assessment of people’s beliefs and behavioral responses to health threats showing that either the individual is in danger control or fear control category [20, 25, 29, 30].

The template was created using Epicollect5, a mobile data-gathering platform. The questionnaire was first developed in English, which had 44 items, and then it was translated into the local language “Amharic” and back to English to ensure consistency and understandability. The data was collected through a face-to-face interviewer-administered questionnaire using Epicollect5. The Interview was held in the local language, Amharic. There were six data collectors (BSC public health). Two days of training were given to data collectors on the data collection tools, use of Epicollect5 software, details of interview techniques, how to approach the participant, the need to respect the rights of participants, and how to maintain confidentiality.

The questionnaire had four parts: the first was Socio-demographic with 8 items, the second was about communication factors which had 3 items; the third part was about individual differences (Self-esteem, Self-control, and Future orientation) with 21 items and the last part was a perception (perceived severity, perceived susceptibility, self-efficacy and response efficacy) with 12 items each of them had 3 items.

### Measurements

Perceived Severity is a belief about the severity of COVID-19. Perceived Susceptibility is a belief of one’s risk of facing COVID-19. Self- Efficacy is a belief in one’s capability to do the suggested response (using a facemask) to avert the threat (COVID-19). Response Efficacy is an acceptance (beliefs) of the effectiveness of the suggested responses (facemask) in decreasing the risk of COVID-19.

Perceived severity, perceived susceptibility, self-efficacy, and response efficacy were measured by 5 points Likert scale (from strongly disagree—strongly agree). After reverse coding the negatively worded statements, the score will be summed up for each respondent. The overall scores of everyone were used to get the mean score. They were treated as continuous variables.

**Behavioral Response**: one of the three outcomes is no response, danger control, or fear control. In this research it was categorized into two danger control responses (it means intended response) and fear control response (it means unintended response) based on the discriminative value (DV). Discriminative value obtained by subtracting the perceived threat score from the perceived efficacy score [20]. **Danger control response** is an intended behavioral response when people believe they are at risk of COVID-19 and believe they can effectively use a facemask to prevent COVID-19. It was a positive score [20].

**Fear control response** is an unintended behavioral response when people are faced with a major and relevant threat but believe that they are unable to use a facemask and/or they believe that the facemask is ineffective. The discriminative value was negative for fear control and zero scores for no response [20].

### Data quality assurance

A pretest was conducted on 5% (32) of the sample size before the actual data collection in the non-selected kebeles of Bahir Dar city administration, which was not included in the study. Finding and experience from the pretest were utilized in modifying the data collection tool and the average time required for the interview was determined, which was 15–20 min. There was regular supervision and support from the data collectors. The reliability test after the final data collection for the four constructs, self-esteem, self-control, and future orientation showed an acceptable internal consistency with a Cronbach alpha of greater than 0.7.

### Data analysis

After the data collection, data were exported to EXCEL from Epicollect5. Finally, EXCEL data were exported to Statistical Product and Service Solutions (SPSS) V.25 for analysis. Descriptive statistics were used to describe the percentage and number of distribution of respondents by each variable. Descriptive summary measures such as mean, and median were computed and the results were presented using texts and tables. Before logistic regression analysis, the assumption was checked, and the data qualified for logistic regression.

Bivariable and multivariable logistic regression analysis was used to identify predictors of behavioral responses. Using the backward likelihood regression variable selection method, independent variables with *P* < 0.25 in the bivariate analysis were entered into the multivariable logistic regression to control the possible effect of confounders. Hosmer-Lame shows Goodness of fit test statistics showed the model as a best-fitted model with a *P*-value of 0.479. Independent variables with *P* < 0.05 and AOR with a 95 percent confidence interval were used in the multivariable model to set the statistically significant level and identify predictors of behavioral response.

## Results

### Socio-demographic characteristics

This study was conducted among 616 participants with a response rate of 97.1%. Of the total participant, 390(63.3%) were females. The mean age of the participants was 32.30 with a standard deviation of 10.64. Concerning participants' educational status 273(44.3%) were college and above. Concerning the participant marital status profile, half of the total 310(50.3%) participants were married. The participants' average monthly income was 4086.94 ± 3793.73 (Table 1).

**Table 1:** Sociodemographic characteristics of respondents in Bahir Dar city, Amhara, Ethiopia 2021 (*N* = 616)

**Variable**	Frequency	Percent (%)
Sex		
Male	226	36.7
Female	390	63.3
Educational status		
Can’t write and read	29	4.7
Write and read	29	4.7
Elementary	67	10.9
High school and preparatory	218	35.4
College and above	273	44.3
Marital Status		
Married	310	50.3
Single	259	42.1
Separated	47	7.6
Occupational Status		
Student	89	14.4
Housewife	138	22.4
Government	108	17.5
Merchant	84	13.6
Private| NGOs	153	24.8
Others	44	7.1
Average monthly income (in ETB)		
(< 1000)	169	27.4
1001–3000	150	24.4
3001–5950	143	23.2
> 5951	154	25.0
Chronic disease		
No	543	88.1
Yes	73	11.9
Number of people live together		
Live alone	54	8.8
Live with 1 & more person	562	91.2

### Communication factor

All the participants 616 (100%) heard about COVID-19. Among the total participants, the most preferred source of information was media 558(26.3%). Television 585(40.9%) was the most preferred channel of the participants (Table 2).

**Table 2:** Distribution of respondents who heard about COVID-19, preferred source of information, and preferred channels in Bahir Dar city, Amhara, Ethiopia 2021 (*N* = 616)

**Variable**	Frequency	Percent (%)
Heard about Corona Virus		
Yes	616	100
The preferred source of information		
Health institution	450	21.2
Media	558	26.3
Religious institution	357	16.8
Friends	240	11.3
Family	301	14.2
Spouse	167	7.9
Others (Facebook, telegram, Internet, YouTube)	11	0.5
Preferred channels		
Television	585	40.9
Radio	285	19.9
Peer discussion	335	23.4
Printed materials	210	14.7
Others (Facebook, telegram, Internet, YouTube)	16	1.1

### Constructs of EPPM

The mean score of perceived threat 22.86 (3.562) was greater than the perceived efficacy 21.46(3.552) (Table 3). This result showed that more people engaged in fear control than danger control. They are engaging either in the defensive avoidance, denial, or reactance phase (Table 4).

**Table 3:** Descriptive statistics of perceived threat, perceived efficacy, self-esteem, self-control, and future orientation in Bahir Dar city, Amhara, Ethiopia 2021 (*N* = 616)

**Variable**	Min	Max	Median	Mean	SD	Scale range	No-of items	Cronbach α
Knowledge	4	15	14.00	13.86	1.556	1–15	15	
Self-esteem	4	20	16.00	14.48	2.921	4–20	4	0.859
Self-control	6	20	14.00	13.76	2.731	4–20	4	0.747
Future orientation	3	15	11.00	10.48	2.308	3–15	3	0.700
Perceived severity	6	15	13.00	12.65	1.996	3–15	3	0.703
Perceived susceptibility	3	15	11.00	10.21	2.409	3–15	3	0.722
Self-efficacy	3	15	10.00	9.63	2.698	3–15	3	0.708
Response efficacy	3	15	12.00	11.83	1.549	3–15	3	0.715
Perceived threat	10	30	23.00	22.86	3.562	12–60		
Perceived efficacy	6	30	22.00	21.46	3.552	12–60

**Table 4:** Distribution of respondents for the items of EPPM constructs in Bahir Dar city, Amhara, Ethiopia 2021 (*N* = 616)

**Variable**	Strongly Disagree No- (%)	Disagree No- (%)	Neutral No- (%)	Agree No- (%)	Strongly agree No- (%)
**Perceived Severity**					
I believe that Corona Virus disease has no cure	4(0.6)	156(25.3)	68(11.0)	261(42.4)	127(20.6)
I believe that Corona Virus disease does not cause death	2(0.3)	15(2.4)	11(1.8)	206(33.4)	382(62.0)
I believe that Corona is a life-threatening disease	2(0.3)	13(2.1)	8(1.3)	222(36.0)	371(60.2)
**Perceived Susceptibility**					
I am at risk of getting the Corona Virus	13(2.1)	108(17.5)	55(8.9)	335(54.4)	105(17.0)
I believe that I will not get infected with Corona Virus disease	6(1.0)	148(24.0)	20(3.2)	306(49.7)	136(22.1)
It is possible that I will have Corona Virus	14(2.3)	229(37.2)	212(34.4)	149(24.2)	12(1.9)
**Self-Efficacy**					
I can use a facemask to prevent getting Corona Virus	27(4.4)	172(27.9)	60(9.7)	303(49.2)	54(8.8)
Facemask is not easy to use to prevent Corona Virus	20(3.2)	137(22.2)	84(13.6)	329(53.4)	46(7.5)
Using facemasks to prevent Corona Virus is convenient	71(11.5)	238(38.6)	37(6.0)	204(33.1)	66(10.7)
**Response Efficacy**					
Facemask works in preventing Corona Virus	3(0.5)	12 (1.9)	19 (3.1)	504(81.8)	78(12.7)
Using a facemask is not effective in preventing Corona Virus	6(1.0)	41(6.7)	106(17.2)	417(67.7)	46(7.5)
If I use a facemask, I am less likely to get Corona Virus	6(1.0)	16 (2.6)	37 (6.0)	438(71.1)	119(19.3)

### Behavioral response to facemask use

Two hundred twenty-nine (37.2%) participants were in the danger control, 27(4.4%) were in the no response and 360 (58.4%) were in the fear control category for facemask use. The participants in the no-response category were added to the fear control category due to very few participants. Overall, 229 (37.2%) participants were in the danger control whereas 387(62.8%) were in the category of fear control responses for facemask use.

### Factors associated with behavioral response to facemask use message

In the bivariate analysis, all variables except sex had a p-value of less than 0.25. They had a significant crude effect or association with the behavioral responses and entered the multivariable analysis. In multivariable analysis, occupational status, number of people living together, self-control, a friend for the preferred source of information, and printed materials for the preferred channel had a significant association with the behavioral response when adjusted to other factors to control the confounding factors with a 95% confidence interval.

The odds of being in the danger control category for face mask use were more likely among residents who were merchants by 3.53 times than students with AOR = 3.53, 95% CI: (1.67–7.46). The odds of being in the danger control category for face mask use was more likely among residents who live with one or more persons by 2.62 times than their counterparts with AOR = 2.62, 95% CI: (1.28–5.39). As a unit increase in self-control sum score, the odds of being in the danger control category were more likely by 14% with AOR = 1.144, 95% CI (1.05–1.25).

The odds of being in the danger control category for face mask use was more likely among residents who chose friends as the preferred source of information by 5.180 times than their counterparts with AOR = 5.180, 95% CI: (3.22–8.33). The odds of being in the danger control category for face mask use was more likely among residents who chose printed materials as the preferred channel by 2.148 times than their counterparts with AOR = 2.148, 95% CI: (1.35–3.43) (Table 5).

**Table 5:** Cross tabulation and multivariable logistic Regression Analysis of factors on Behavioral Response among residents in Bahir Dar city, Amhara, Ethiopia 2021 (*N* = 616)

Factors	Behavioral Response		OR	
	Fear Control	Danger Control	COR (95% CI)	AOR (95% CI)
Age			1.011(1.00–1.03)	1.003(0.98–1.03)
Educational Status				
Can’t write & read	24(3.9%)	5(4.7%)	1	1
Write & read	14(2.3%)	15(2.4%)	6.696(1.86–24.14)	2.711(0.61–12.04)
Elementary	46(7.5%)	21(3.4%)	2.853(0.88–9.24)	1.345(0.36–5.02)
High school and preparatory	134(21.8%)	84(13.6%)	3.918(1.32–11.66)	1.734(0.49–6.09)
College and above	169(27.4%)	104(16.9%)	3.906(1.32–11.54)	1.599(0.44–5.78)
Marital status				
Married	184(29.9%)	126(20.5%)	1	1
Single	170(27.6%)	89(14.4%)	0.752(0.53–1.06)	1.726(0.92–3.24)
Separated	33(5.4%)	14(2.3%)	0.69- (0.36–1.31)	1.980(0.87–4. 50)
Occupation				
Student	60(9.7%)	29(4.7%)	1	1
Housewife	100(16.2%	38(6.2%)	0.786(0.44–1.40)	1.056(0.53–2.10
Government	68(17.6%)	40(17.5%)	1.217(0.67–2.20)	1.088(0.54–2.21)
Merchant	37(6.0%)	47(7.6%)	2.628(1.42–4.87)	3.533(1.67–7.46) ^a^
Private & NGOS	95(15.4%)	58(9.4%)	1.335(0.77–2.31)	1.378(0.72–2.65)
Others	29(4.7%)	15(2.4%)	1.070(0. 50–2.30)	1.507(0.60–3.79)
Average monthly income				
< 1000	108(17.5%)	61(9.9%)	1	1
1001–3000	101(16.4%)	49(8%)	0.859(0.54–1.37)	1.000(0.51–1.97)
3001–5950	103(16.7%)	40(6.5%)	0.688(0.43–1.11)	0.671(0.33–1.38)
> 5951	75(12.2%)	79(12.8%)	1.865(1.20–2.91)	1.043(0.49–2.23)
Chronic diseases				
No	352(57.1%)	191(31.0%)	1	1
Yes	35(5.7%)	38(6.2%)	1.764(1.08–2.884)	0.906(0.44–1.86)
People live together				
Live alone	38(6.2%)	16 (2.6%)	1	1
Live with 1 & more person	349(56.7%)	213(34.6%)	1.449(0.79–2.66)	2.624(1.28–5.39)^a^
Knowledge			1.292(1.14–1.47)	1.143(0.99–1.31)
Self-esteem			1.171(1.10–1.25)	1.024(0.95–1.11)
Self-control			1.340(1.24–1.45)	1.144(1.05–1.25)^a^
Future orientation			1.296(1.19–1.41)	1.071(0.97–1.18)
The preferred source of information is health institution				
No	120(19.5%)	46(7.5%)	1	1
yes	267(43.3%)	183(29.7%)	1.788(1.21–2.64)	1.111(0. 66–1.86)
The preferred source of information is media				
No	46(7.5%)	12(1.9%)	1	1
Yes	341(55.4%)	217(35.2%)	2.186(1.15–4.15)	1.016 (0.45–2.27)
The preferred source of information is religiousinstitution				
No	204(33.1%)	55(8.9%)	1	1
Yes	183(29.7%)	174(28.2%)	3.295(2.30–4.72)	1.306(0.80–2.13)
The preferred source of information is friends				
No	308(50.0%)	68(11.0%)	1	1
Yes	79(12.8%)	161(26.1%)	8.581(5.91–12.46)	5.180(3.22–8.33)^a^
The preferred source of an information is family				
No	257(41.7%)	58(9.4%)	1	1
Yes	130(33.6%)	171(74.7%)	5.441(3.79–7.81)	1.026(0.52–2.04)
The preferred source of an information is a spouse				
No	330(53.6%)	119(19.3%)	1	1
Yes	57(9.3%)	110(17.9%)	5.352(3.65–7.84)	1.490(0.87–2.56)
Preferred channel is television				
No	26(4.2%)	5(0.8%)	1	1
Yes	361(58.6%)	224(36.4%)	3.227(1.22–8.52)	1.690(0.54–5.31)
Preferred channel is radio				
No	249(40.4%)	82(13.3%)	1	1
Yes	138(22.4%)	147(23.9%)	3.138(2.23–4.41)	0.696(0.41–1.18)
Preferred channel is Peer discussion				
No	208(33.8%)	73(11.9%)	1	1
Yes	387(29.1%)	229(25.3%	2.560(1.82–3.61)	0.961(0.57–1. 610)
Preferred channel is printed materials				
No	310(50.3%)	96(15.6%)	1	1
Yes	77(12.5%)	133(21.6%)	5.212(3.64–7.47)	2.148(1.35–3.43) ^a^

^**a**^statistically significant at α = 0.05

The final model explains 76.9% of predictions of the outcome variable (behavioral response) with a goodness of fit of the model (× 2/df = 7.543/8, *p*-value = 0.479).

## Discussion

Starting from the outbreak of COVID-19, many people died, and it causes severe morbidity around the world. It is causing social, psychological, and socio-economic impacts all over the world. Behavioral responses to COVID-19 prevention messages can control the transmission patterns of disease and the number of new cases.

The overall finding of the study indicated that 37.2% (33.3%-41.1%) of participants were in the danger control behavioral response. This finding was lower than studies conducted among healthcare workers in North Shoa [16], the Ethiopian online survey [19], and Iran [17, 31, 32]. This discrepancy might be due to the variation of the data collection period even if evidence indicates that as COVID-19 progresses, people will have a greater awareness of the health risks caused by COVID-19 and engage in the recommended behavior [33].

Another difference might be due to development status, perceived threat, and perceived efficacy levels. According to the EPPM, high-perceived efficacy with high-perceived threat and high-perceived efficacy with low perceived threat leads to danger control while high-perceived threat with low perceived efficacy leads to a fear control response [20, 22].

There may be also a difference in the individuals’ engagement behavior; there is a greater tendency to engage in preventive behavior among some people than others [34]. In addition, it might be due to differences in attitude, intention to use a facemask, and level of education since their study focus only on the educated person. As Kim Witte in the effective health risk message: a step-by-step guide stated that people in the danger control response have higher attitudes, intentions, and recommended behaviors [20].

Being Merchants in occupation was a positive predictor of behavioral response. In this study, merchants were more likely to be in danger control than students. This is similar to the study done in Iran [31] and the United States [34]. This might be due to merchants having frequent travel and contact with many people.

In this study, the number of people who live together had a positive association with the behavioral response. A person who lives with one or more people was more likely to be in the danger control category. This is similar to a study conducted in China [35], Greater Toronto [36], and the United States [34]. This might be due to the pandemic nature of the disease, fear of acquiring the disease, and fear of transmission within the house. According to EPPM, fear motivates action or engagement in the recommended behaviors which leads to the danger control response [20, 22].

Friends as a preferred source of information had a positive significant association with the behavioral response. In this study people who choose friends as a preferred source of information were more likely to be in danger control than people who do not choose friends. This finding contradicts a study done on an online survey in Ethiopia [19]. This might be due to the trust among friends, sharing of ideas, and the willingness to communicate with friends.

Printed materials as a preferred channel were positive predictors of behavioral response. In this study people who choose printed materials as their preferred channel were more likely to be in danger control than people who don't choose printed materials. The reason might be due to the transmission of facts related to facemask and their importance to prevent COVID-19. This is different from the study done in Israel [37]. This might be due to variations in the study settings and perceived efficacy levels.

Self-control had a positive association with the behavioral response. This finding is in line with the studies done in China and the U.S [38, 39]. This might be because people with high self-control can accept the prevention message and use a facemask. The more people have self-regulatory behavior they are more likely to be in the danger control response [20, 40].

Finally, the Authors would like to report the Limitation of the study in that it was a cross-sectional study that does not show cause and effect relationship. The face-to-face interview might have social desirability bias. It assessed household average monthly income so people may not tell us their income accurately. This study was quantitative research that did not explore why people were present in the fear control category.

## Conclusions

In this study the danger control response was low. Perceived efficacy is lower than a perceived threat. Occupational status, the number of people who live together, self-control, a friend for the preferred source of information, and printed materials for the preferred channel were predictors of behavioral response for facemask use.

To improve face mask use behavior and for controlling COVID-19, the study findings suggest strategies like:

Policymakers should consider students and people who live alone. This can be achieved by creating access, the ability to wear a facemask, and a suitable environment at school. Message developers should use a friendly person to transmit messages and should prepare printed materials. Messages which focus on perceived efficacy toward facemask use without ignoring the perceived threat and self-control should be designed. For the future researcher, it is better to triangulate the quantitative with the qualitative findings.

## Supplementary Information

**Additional file 1:** **Supplementary figure S1.** Conceptual framework of behavioral responses for facemask use messages to prevent COVID 19 among residents of Bahir Dar City.

**Additional file 2:** **Supplementary tool S2.** Data collection tool English version.

## Data Availability

The datasets used and/or analyzed during the current study are available from the corresponding author upon reasonable request.

## Abbreviations

COVID-19: Coronavirus disease 2019
EPPM: Extended parallel process model
RCCE: Risk communication and community engagement
SARS: Severe acute respiratory syndrome
SARS, COV- 2: Severe acute respiratory syndrome Coronavirus two
SPSS: Statistical product and service solutions
WHO: World health organization
IRB: Institution Review Board
CI: Confidence interval
AOR: Adjusted odd ratio
