# Supplementary figures and images for: Behavioral responses for facemask use messages to prevent COVID-19 among residents of Bahir Dar City, Ethiopia: an application of extended parallel process model
Source: BMC Public Health. 2022 Dec 22;22:2409. doi: 10.1186/s12889-022-14872-5 (PMC9773474; doi:10.1186/s12889-022-14872-5)

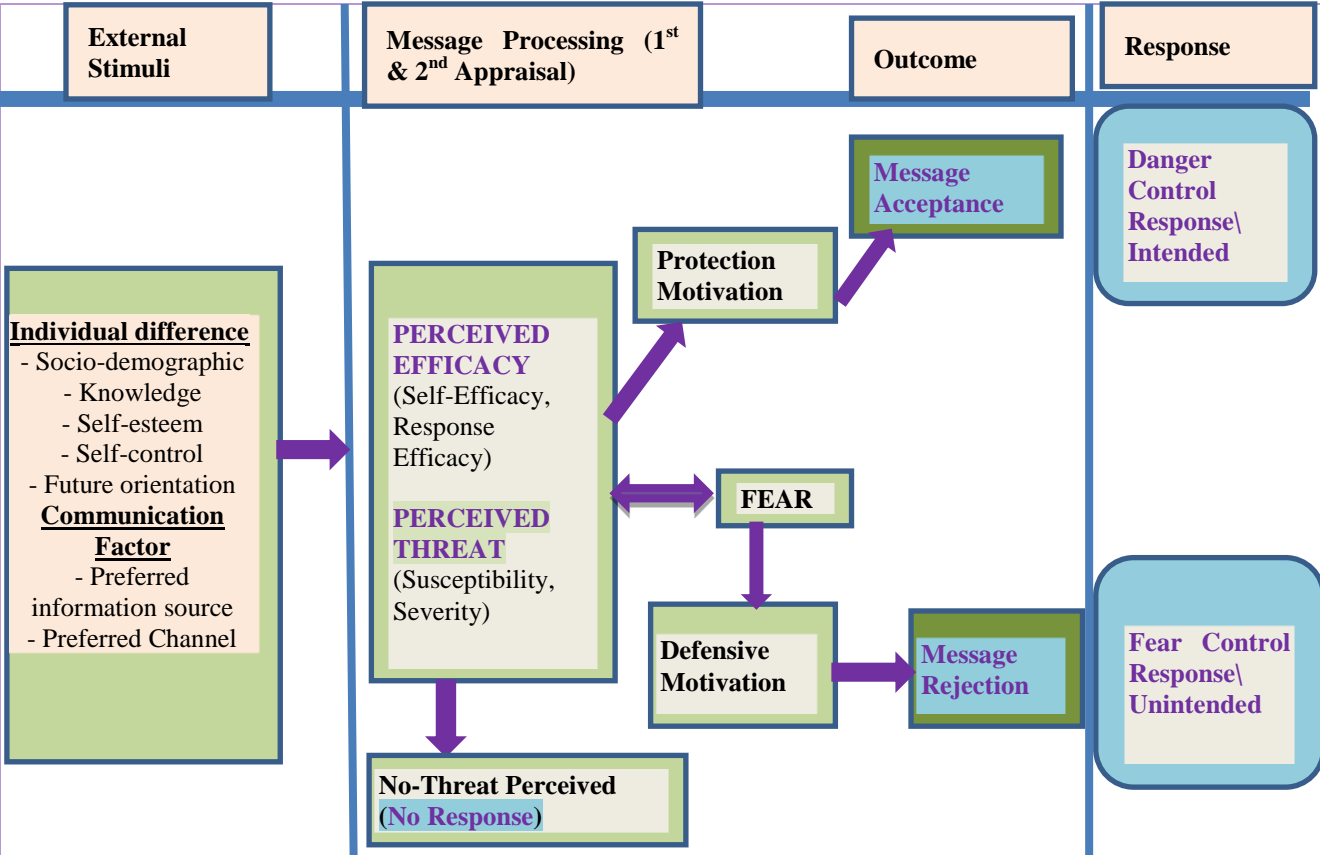

(Adapted from: Kim Witte (2001), Effective Health risk messages a step-by-step guide

Supplement: Supplementary file 1 — Additional file 1: Supplementary figure S1. Conceptual framework of behavioral responses for facemask use messages to prevent COVID 19 among residents of Bahir Dar City. [file 12889_2022_14872_MOESM1_ESM.pdf]
