# Supplementary material for: Behavioral responses for facemask use messages to prevent COVID-19 among residents of Bahir Dar City, Ethiopia: an application of extended parallel process model
Source: BMC Public Health. 2022 Dec 22;22:2409. doi: 10.1186/s12889-022-14872-5 (PMC9773474; doi:10.1186/s12889-022-14872-5)
Supplement: Supplementary file 2 — Additional file 2: Supplementary tool S2. Data collection tool English version. [file 12889_2022_14872_MOESM2_ESM.pdf]

Bahir Dar University College of Medicine and Health Science School of Public Health

Department of health promotion and behavioral Sciences

CONSENT FORM AND INFORMATION SHEET

Hello! Dear sir / madam,

Bahir Dar University, Department of health promotion and behavioral sciences conducting a research on "Behavioral responses for the facemask use messages to prevent COVID-19 among residents of Bahir Dar city, Amhara, Ethiopia". Permission was obtained from the research and ethical committee of Bahir Dar University and Bahir Dar city administration to conduct this study.

The objective of this study is to assess behavioral responses for the facemask use messages to prevent COVID-19 among residents of Bahir Dar city. The main part of the study involves collecting information from 634 respondents like you. You are selected randomly. We are kindly requesting you to answer the questions that we have prepared for you. The interview will be held in Amharic for about 15-20 minutes. One person will be asked once at a time.

We assure all information gathered during the course of the study will be kept completely secure (confidential). All the information that you are going to deliver to us will be coded for anonymity. You have the right to raise questions at any time for clear understanding.

There is no incentive or payment for participating in this research. Participation is voluntary; you do not have to answer any questions that you do not want to answer. You may end to participate in the study at any time you want without giving a reason and without fear. No force or harm will be occurred. However, your honest answers to these questions will help us to better understand people's perceptions about COVID-19 and behavioral responses to the facemask use messages for better decision-making and interventions.

1. Would you be willing to participate? 1. Yes 2. No
2. Interviewer name Date

_____________________ _________________

If you have questions about the research, contact Tenagnework Eseyneh (Email Adress: tenagneworke1@gmail.com, Mobile phone no- = +251-949173682)

QUESTIONAIRE

PART 1: Socio demographic Data

CODE	VARIABLE	ANSWER
101	Sex	Female<br>Male
102	Age	________________________
103	Educational Status	Can't write and read<br>write and read<br>Elementary<br>High school and preparatory<br>College and above
104	Marital Status	Married<br>Single<br>Divorced<br>Husband | wife died
105	Occupational Status	Student<br>Housewife<br>Government<br>Merchant<br>Private| NGOs<br>Others specify…
106	Average monthly income (in ETB)	_____________
107	Do you have Chronic disease?(Hypertension, Diabetes Mellitus, Heart Failure, Asthmatic …)	Yes<br>No
108	How many people live With you?	______________

Part 2 Communication Factor

The following statements are about your source of information and preference of COVID-19 message, source, and channel Please give your answer according to the statements (Yes or No).

CODE	Variable		Yes	No
201	Have you heard about Corona Virus?			

202	Preferred sources of Information<br><br>(more than one answer is possible)	Health institution		
		Media		
		Religious institutions		
		Friends		
		Family		
		Spouse		
		Other specify…		
203	Preferred channels<br><br>(more than one answer is possible)	Television		
		Radio		
		Peer discussion		
		Printed materials(leaflets,<br><br>posters, poems)		
		Others specify…		

PART 3 Miscellanies scales: Knowledge, Self-esteem, Self-control, and Future orientations

The following are statements about individual characteristics, Please give your answer according to your agreement to the statements according to the scale.

Just please give your honest opinion.

CODE	Knowledge related to COVID-19	Yes	No	I don't know
301	Is Coronavirus disease caused by a newly discovered			

	coronavirus?				
302	Which one is the main clinical symptom of COVID-19?	Fever			
		Fatigue			
		Continuous dry cough			
		shortness of breath			
303	Is COVID-19 a transmissible disease/contagious?				
304	Does the COVID-19 virus spread primarily through droplets of saliva or discharge from the nose when an infected person coughs or sneezes?				
305	Are Elderly people or people with chronic health conditions developing severe conditions if infected with COVID-19?				
306	Do People infected with the COVID-19 virus without any symptoms will not infect others?				
307	Currently, Is there an effective drug to cure COVID-19?				
308	Does early diagnosis of COVID-19 improve the treatment?				
309	Is COVID-19 being a preventable disease?				
310	Which one can reduce the risk of COVID-19 infection?	Using facemask			
		Handwashing with soap			
		physical distancing			

1= Strongly Disagree, 2= Disagree, 3= Neutral, 4= Agree, 5= Strongly Agree

CODE	Items	1	2	3	4	5

	Self-Esteem					
311	You believe you have satisfied with yourself.					
312	You think you have good qualities.					
313	Sometimes you think you are not good at all.					
314	You wish I were someone else.					
	Self-control					
315	You think carefully about all your choices.					
316	You will do what you think good for the moment.					
317	Sometimes you like breaking rules.					
318	You do it quietly without thinking.					
	Future orientation					
319	You will enjoy today because you may die tomorrow.					
320	You try not to think about your future.					
321	You try to save money for other days.					

PART 4 Perceptions about COVID-19

The following statements are about your perception of COVID-19 and the prevention method. Please give your answer according to your agreement to the statements according to the scale.

1= Strongly Disagree, 2= Disagree, 3= Neutral, 4= Agree, 5= Strongly Agree

CODE	ITEM	1	2	3	4	5
	PERCEIVED THREAT					

	Perceived Severity					
401	I believe that Corona Virus disease has no cure.					
402	I believe that Corona Virus disease does not cause death.					
403	I believe that Corona is a life-threatening disease.					
	Perceived Susceptibility	1	2	3	4	5
404	I am at risk of getting Corona Virus.					
405	I believe that I will not get infected with Corona Virus disease.					
406	It is possible that I will have Corona Virus.					
	PERCEIVED EFFICACY					
	Self-Efficacy	1	2	3	4	5
407	I can use a facemask to prevent getting Corona Virus.					
408	Facemask is not easy to use to prevent Corona Virus.					
409	Using facemasks to prevent Corona Virus is convenient.					
	Response Efficacy	1	2	3	4	5
210	Facemask works in preventing Corona Virus.					
211	Using a facemask is not effective in preventing Corona Virus.					
212	If I use a facemask, I am less likely to get Corona Virus.					
